# Mucociliary clearance is impaired in small airways of cystic fibrosis pigs

**DOI:** 10.1152/ajplung.00010.2024

**Published:** 2024-08-06

**Authors:** Carley G. Stewart, Brieanna M. Hilkin, Nicholas D. Gansemer, Ryan J. Adam, David W. Dick, John J. Sunderland, David A. Stoltz, Joseph Zabner, Mahmoud H. Abou Alaiwa

**Affiliations:** ^1^Department of Biomedical Engineering, University of Iowa, Iowa City, Iowa, United States; ^2^Wisconsin National Primate Research Center, University of Wisconsin-Madison, Madison, Wisconsin, United States; ^3^Department of Internal Medicine, Roy J. and Lucille A. Carver College of Medicine, University of Iowa, Iowa City, Iowa, United States; ^4^Department of Radiology, Roy J. and Lucille A. Carver College of Medicine, University of Iowa, Iowa City, Iowa, United States; ^5^Department of Molecular Physiology and Biophysics, Roy J. and Lucille A. Carver College of Medicine, University of Iowa, Iowa City, Iowa, United States; ^6^Pappajohn Biomedical Institute, Roy J. and Lucille A. Carver College of Medicine, University of Iowa, Iowa City, Iowa, United States

**Keywords:** cystic fibrosis, mucociliary clearance, mucus, mucus inspissation of respiratory tract, positron emission tomography-computed tomography

## Abstract

Cystic fibrosis (CF) is a genetic disorder characterized by recurrent airway infections, inflammation, impaired mucociliary clearance, and progressive decline in lung function. The disease may start in the small airways; however, this is difficult to prove due to the limited accessibility of the small airways with the current single-photon mucociliary clearance assay. Here, we developed a dynamic positron emission tomography assay with high spatial and temporal resolution. We tested that mucociliary clearance is abnormal in the small airways of newborn cystic fibrosis pigs. Clearance of [^68^Ga]-tagged macroaggregated albumin from small airways started immediately after delivery and continued for the duration of the study. Initial clearance was fast but slowed down a few minutes after delivery. Cystic fibrosis pigs’ small airways cleared significantly less than non-CF pigs’ small airways (non-CF 25.1 ± 3.1% vs. CF 14.6 ± 0.1%). Stimulation of the cystic fibrosis airways with the purinergic secretagogue uridine-5′-triphosphate (UTP) further impaired clearance (non-CF with UTP 20.9 ± 0.3% vs. CF with UTP 13.0 ± 1.8%). None of the cystic fibrosis pigs treated with UTP (*n* = 6) cleared more than 20% of the delivered dose. These data indicate that mucociliary clearance in the small airways is fast and can easily be missed if the assay is not sensitive enough. The data also indicate that mucociliary clearance is impaired in the small airways of cystic fibrosis pigs. This defect is exacerbated by stimulation of mucus secretions with purinergic agonists.

**NEW & NOTEWORTHY** We developed a novel positron emission tomography scan assay with unprecedented temporal and spatial resolution to measure mucociliary clearance in the small airways. We proved a long-standing but unproven assertion that mucociliary clearance is inherently abnormal in the small airways of newborn cystic fibrosis piglets that are otherwise free of infection or inflammation. This technique can be easily extended to other airway diseases such as asthma, idiopathic pulmonary fibrosis, or chronic obstructive pulmonary disease.

## Graphical abstract

**Figure:**
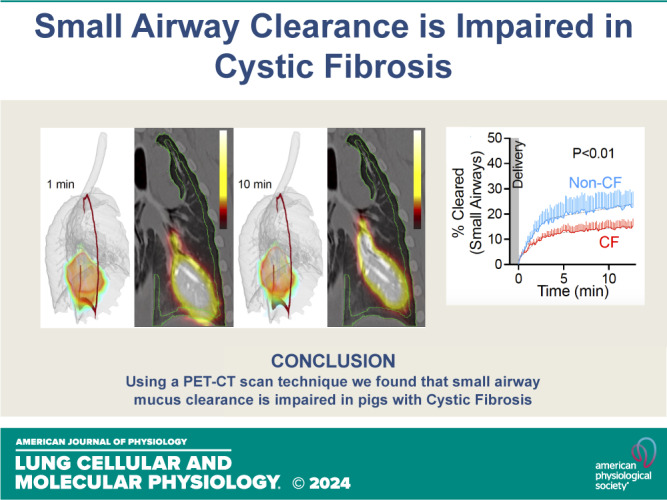

## INTRODUCTION

Cystic fibrosis (CF) is a genetic disease caused by mutations in the cystic fibrosis transmembrane conductance regulator (*CFTR*) gene with high morbidity and mortality arising from airway involvement (1–3). Although previous observations in autopsy studies (4), spirometry studies (5), multiple breath washout studies (6), and computed tomography (CT) and hyperpolarized ^3^He MRI studies (7) have hinted at the role of small airways (8, 9) in CF pathogenesis, this hypothesis remained untested due to the lack of a suitable animal model and technical difficulties accessing the small airways (2).

The mammalian airways branch in a dichotomous pattern for ∼22 generations (10, 11). With each generation of branching, a parent airway splits into ∼2 smaller and shorter daughter airways (12, 13). The small airways are distinctively different from the rest of the conducting airways. They contribute the least to total airway resistance (14). They have a distinct cuboidal appearance, lack submucosal glands, lack complete cartilage rings, and their secretory cells express surfactant protein D (SPD) (15). SPD has a role in reducing airway surface liquid (ASL) surface tension and may help prevent airway closure on expiration (16). In young piglets, the airways with a diameter less the 200 µm have these characteristics (17).

We developed a porcine animal model of CF with targeted disruption of the *CFTR* gene (18). At birth, these pigs show no signs of infection or inflammation; yet their airways are unable to transport particles or kill bacteria (19–21). As they grow older, they develop the hallmarks of human CF airway disease, including spontaneous bacterial infection, neutrophilic inflammation, and mucus plugging (18). We wanted to test the hypothesis that mucociliary clearance is impaired in the small airways of newborn CF piglets that are free of infection or inflammation. However, the current single-photon mucociliary clearance assays are lacking in temporal and spatial resolution. To circumvent these limitations, we developed a novel dynamic positron emission tomography (PET) technique to measure mucus transport in the small airways (22).

## MATERIALS AND METHODS

### Animals

We previously reported the production of *CFTR^−/−^* pigs (23). Newborn piglets were obtained from Exemplar Genetics. Animals were studied 8–15 h postbirth. Sedation was with ketamine [20 mg/kg, intramuscular (im); Phoenix Pharmaceutical, Inc.] and acepromazine (2 mg/kg, im; Phoenix Pharmaceutical, Inc.) and anesthesia was maintained with dexmedetomidine (iv) (10 mg/kg/h; Accord Healthcare, Inc.). Euthanasia was with Euthasol (iv) (Virbac).

### Micro-CT

To determine the distance from the pleura that contains most of the small airways with a diameter of less than 200 µm, we scanned a tracheal lobe at 20 cmH_2_O with a Siemens micro-CAT II scanner (Siemens, Pre-Clinical Solutions; Knoxville, TN) and reconstructed with an isotropic voxel spacing of 0.028 mm. The size and location of small airways should be the same throughout the lungs. We segmented the tracheal lobe and the airways in ITK-SNAP (24) using region growing thresholding [Hounsfield Unit (HU) < −700 for airways and HU < −200 for lung parenchyma]. We iteratively dilated the boundary of the lung to grow a three-dimensional (3-D) shell and determined the depth of the shell that enclosed most of the small airways.

### In Vivo Mucociliary Clearance Assay

We used macroaggregated albumin (MAA) labeled with a positron-emitting isotope and monitored the movement with dynamic PET scan imaging. Inspired by the use of Tc-99m macroaggregated albumin (MAA) to measure tracheal mucus velocity (25), we radiolabeled MAA with gallium-68. Gallium-68 was used because it is readily available, has a convenient short half-life of 68 min, decays into positrons of sufficient energy to create high-quality images, easy to label with MAA (26), and facilitates the use of high temporal and spatial resolution, quantitative PET/CT rather than single-photon imaging. Gallium-68 was attached to MAA with 15–30 μm predicted particle size (27). Gallium-68 was obtained by eluting a germanium-68/gallium-68 generator and applying a commercial MAA labeling kit (Pulmotech MAA; Curium US, Maryland Heights, MO). [Ga-68] MAA particles (∼15 to 30 μm, 15–100 mCi) were delivered using 1.5-mm polyethylene tubing (Intramedic; Parsippany, NJ) introduced into the distal airways of sedated newborn pigs. The piglets were intubated with a size 3.0 uncuffed endotracheal tube and allowed to breathe spontaneously. The catheter was inserted via the endotracheal tube and advanced into the airways until resistance was met. Then, 500 µL of the radiolabeled tracer followed by 500 µL of air were injected. Both the catheter and the endotracheal tube were immediately retrieved.

### PET/CT Imaging

We acquired PET data in a continuous list mode for 15 min on a Discovery Molecular Imaging Digital Ready PET/CT scanner (GE Healthcare). This machine has a sensitivity of 7.3 cps/kBq and spatial resolution full-width-half-maximum radial 5.5 mm, tangential 4.5 mm, and axial 6 mm at 10 cm from the center of the field of view (28). We also acquired a high-resolution CT scan at the same time on spontaneously breathing animals with no specific consideration to capture at end-inspiration or end-expiration. We used these images for anatomical references. We binned the data into 10-s timeframes to reconstruct the time-lapsed PET images.

### Data Analysis

Because the transfer function of the PET scan camera is much larger than the size of newborn pigs and the tissue motion from breathing can create a blurring effect, we used two methods to quantify the PET signal in the distal airways. We reconstructed and time-binned the images on the GE PET scan acquisition software. We converted the DICOM files into volumetric datasets using dcm2niix (29). We segmented and created label maps for the airways and lung parenchyma in 3-D Slicer (30) using region growing thresholding (HU < −700 for airways, HU < −200 for lung parenchyma). To extract the PET signal localized to a 3-D shell that surrounds the lungs, we started with a segmentation of the lung parenchyma. In the 3-D Slicer Segment Editor module, we filled the gaps in the mesh using the closing (fill holes) method, created the 1.5-mm-thick shell with the Hollow function, and extracted the PET signal within the 3-D shell volume. To extract the PET signal localized within the small airways, we started with a segmentation of the airways. To find the centerline of the airways, we applied a 3-D thinning algorithm (31) and fitted a cubic spline to the centerline. We extrapolated the line to the pleural surface. We uniformly spaced perpendicular planes to the centerline. Each plane sectioned the airway volume into a thin disk. We extracted the PET signal from each disk. We wrote custom scripts in Python to automate this process. These scripts depended on the following Python libraries: numpy 1.26.3 (32), nibabel 5.2.0 (33), NURBS (geomdl) 5.3.1 (34), and SimpleITK 2.3.1 (35–37).

### Statistical Analysis

**Figure 1.**
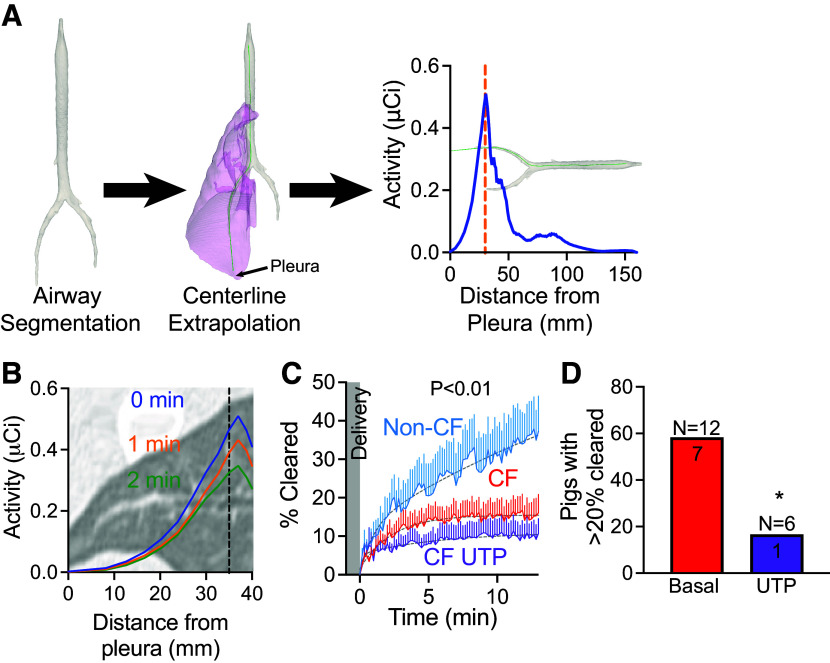
Mucociliary clearance is impaired in the distal airways of CF pigs. *A*: PET signal (activity in µCi) as we move away from the pleura. *B*: clearance from distal airways is fast (in minutes). *C*: percent change in the activity of delivered dose (%Cleared) from distal airways as a function of time for non-CF (blue), CF (red), or CF with UTP (purple). Lines represent means ± SE. Gray dashed line represents two-phase exponential fit to the data. *P* value < 0.01 by *F* test. *D*: number of CF pigs with clearance greater than 20% at the end of the study. CF in red and CF with UTP in purple. CF, cystic fibrosis; PET, positron emission tomography. *P < 0.01 by Fisher’s exact test.

**Figure 2.**
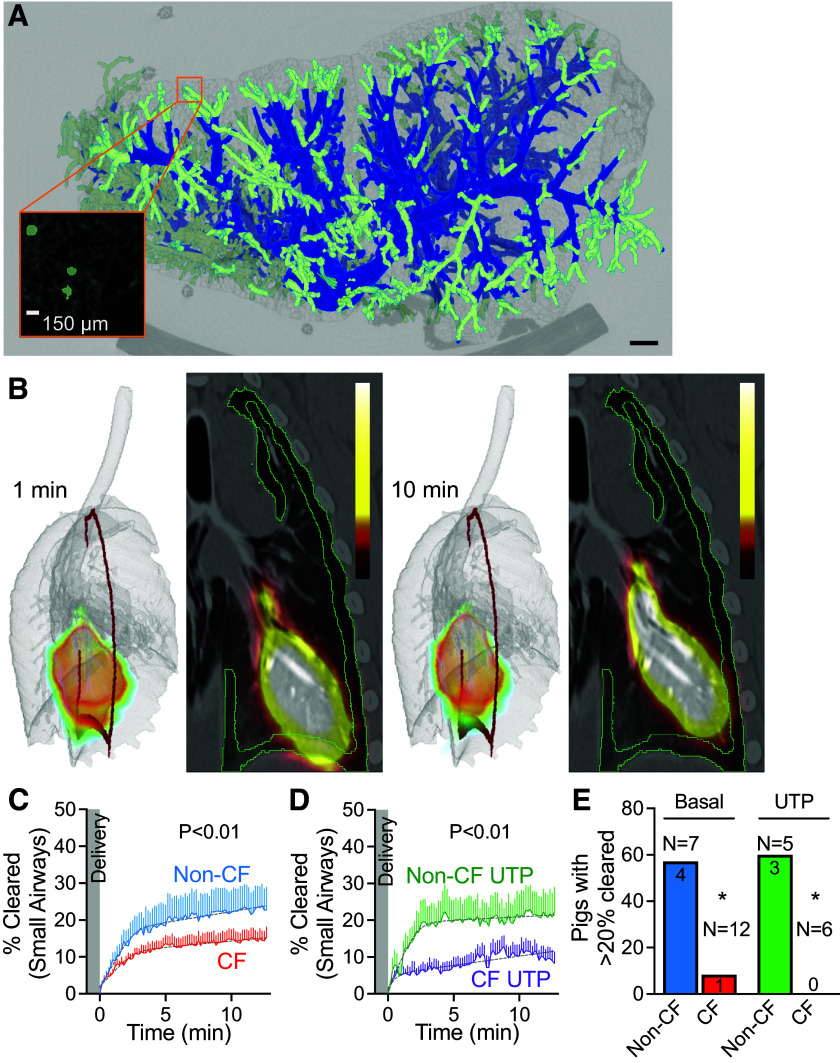
Mucociliary clearance is impaired in the small airways of CF pigs. *A*: micro-CT of the porpoise lobe of newborn pig. Small airways (less than 200 µm in diameter, light green) are present within a 1.5-mm 3-D shell inscribed in the outer lung (*inset*). Scale = 150 µm. *B*: images are composite PET signal superimposed on volumetric reconstruction of the lungs (*left*) and a 2-D planar projection (*right*). Mucociliary is immediate and fast. Red (*left*) and green lines (*right*) represent cross section of a 1.5-mm 3-D shell. Scale = 0 µCi–10 µCi. *C* and *D*: percent change in the activity of delivered dose (%Cleared) from small airways as a function of time in non-CF vs. CF newborn pig airways. *C*: basal (non-CF in blue and CF in red). *D*: UTP stimulation (non-CF in green and CF in purple). Lines represent means ± SE. Gray dashed line represents two-phase exponential fit to the data. *P* value < 0.01 by *F* test. *E*: number of pigs with clearance greater than 20% at the end of the study. Non-CF at basal condition in blue, CF at basal condition in red, non-CF after UTP stimulation in green, and CF after UTP stimulation in purple. CF, cystic fibrosis; PET, positron emission tomography; 3-D, three-dimensional, 2-D, two-dimensional. *P < 0.01 by Fisher’s exact test.

Differences were considered statistically significant at *P* < 0.01. All analyses were completed in GraphPad Prism v9.5.1 (GraphPad Software, La Jolla, CA). Data are presented as mean ± SE and are indicated by bars. For %Cleared in Fig. 1*C* and Fig. 2, *C* and *D*, we modeled the radiotracer clearance with a simple (Occam’s Razor) 2-phase exponential model (3 parameters, all curves start at 0% clearance). We used the extra sum-of-squares *F* test to reject the null hypothesis that the simpler model, one fitted curve explains all the variability in the data. The *P* value explains how rare is the coincidence that random scatter explains all the variability in the data. We used Fisher’s exact test to calculate the *P* value for contingency data in Figs. 1*D* and 2*E*.

### Study Approval

All animal studies protocols were submitted and approved by The University of Iowa Animal Care and Use Committee.

## RESULTS

To investigate small airway clearance, we used two complementary approaches. We first extended the main airway by extrapolating the airway centerline to the pleura. With this approach, we minimized the signal from the parenchyma, but we limited our analysis to a small number of distal airways. Second, we analyzed the clearance of the peripheral 1.5-mm three-dimensional (3-D) shell where most of the airways are smaller than 200 µm.

### Mucus Transport Is Impaired in the Distal Airways of CF Pigs

The high-resolution CT scan in our study of newborn pigs had limited resolving power to 2 mm in diameter airways. Therefore, we extended the centerline of the main lobar airway by extrapolation and sampled the radiotracer signal from a 2-mm-diameter cylinder that extends to the visceral pleura (Fig. 1*A*). The radiotracer started to clear from the distal airways of a non-CF pig, within one minute of deposition (Fig. 1*B*). Clearance of the radiotracer from the distal airways followed a two-phase process, with an immediate brisk phase of clearance within the first 5 min after delivery followed by slower clearance (Fig. 1*C*). In non-CF pigs, ∼40% of the signal cleared within the 12-min acquisition period and never reached a plateau. In CF pigs, clearance was impaired and further reduced after stimulation with the purinergic secretagogue uridine-5′-triphosphate (UTP) (CF 15.5 ± 0.7% vs. CF with UTP 11.1 ± 2.4%) (Fig. 1*C*). Studies using planar single-photon mucociliary clearance report total lung airway clearance of ∼20% of the delivered dose after 2 h (38). In this study, all non-CF pigs cleared more than 20% in 12 min and continued to clear by the end of the study. However, only 7 out of 12 CF pigs (58.33%, *n* = 12) and only 1 CF pig out of 6 treated with UTP (16.67%, *n* = 6), cleared more than 20% of the delivered dose in 12 min (Fig. 1*E*).

### Mucus Clearance Is Impaired in the Small Airways of CF Pigs

To measure clearance from the small airways, we inscribed a 3-D shallow shell within the outer lung. A shell with a depth of 1.5 mm enclosed most of the airways with a diameter of less than 200 µm, as demonstrated in a micro-CT reconstruction of the porpoise lobe (Fig. 2*A*) (39). We measured mucociliary clearance out of this thin 3-D shell (Fig. 2*B*). Clearance started within a minute after delivery and continued for the duration of the study (Fig. 2, *C* and *D*). Early clearance rates were fast but slowed down a few minutes after delivery (Fig. 2, *C* and *D*). CF pigs cleared significantly less than non-CF (non-CF 25.1 ± 3.1% vs. CF 14.6 ± 0.1%) (Fig. 2*C*). Moreover, only 1 out of 12 CF pigs cleared more than 20% of the delivered dose within 12 min, suggesting that clearance in CF small airways is worse than that seen in distal lung airways (Fig. 1*D*).

### Purinergic Stimulation Further Impairs Small Airways Clearance in CF Pigs

Purinergic stimulation is predicted to stimulate mucus secretion and chloride secretion (TMEM16A, transmembrane protein 16A or anoctamin1) and predicted to increase ciliary beat frequency (40–42). All these changes are predicted to increase mucociliary clearance. After UTP stimulation, small airway clearance remained impaired in CF compared with non-CF (non-CF with UTP 20.9 ± 0.3% vs. CF with UTP 13.0 ± 1.8%) (Fig. 2*D*). Surprisingly, UTP stimulation made clearance worse in CF pigs (CF basal 14.6 ± 0.1% vs. CF with UTP 13.0 ± 1.8%, *P* < 0.01). There was no change in clearance in non-CF pigs after UTP stimulation. As a result, most of the non-CF pigs (57%, *n* = 7) cleared more than 20% of the delivered dose at the end of the study. However, only one CF pig at the basal condition (8%, *n* = 12) and none of the CF pigs treated with UTP (0%, *n* = 6) cleared more than 20% of the delivered dose at the end of the study (Fig. 2*E*).

## DISCUSSION

The submucosal gland duct opening is the site where mucus strands with abnormal biophysical properties get stuck and impede mucociliary transport in the CF large airways (20, 21). The small airways lack submucosal glands (17), so it is conceivable that mucociliary clearance is normal in the CF small airways. Our data show the opposite and indicate that mucociliary clearance is also impaired in the pristine small airways of newborn CF pigs before the onset of infection or inflammation. This defect is accentuated by the stimulation of mucus secretion with purinergic agonists.

In autopsy studies in CF, mucus plugging or mucoviscidosis in the small airways is a common pathologic finding (43). However, the origin of mucus in these mucus plugs is debated. One possibility is that the mucus is secreted in the small airways but because it is not properly cleared, it piles up into mucus plugs (8, 44). Another possibility is that the mucus originates in the large airways and is deposited in the small airways by mere gravity (45). In prior work in newborn CF pigs, we described an unusual abrupt backward movement of mucus strands secreted by submucosal glands in the large airways (21). This behavior may offer an explanation. The mucus strands snap, ricochet, and lodge in the small airways. Our current data, however, support the former explanation and provide insight into the origin of mucus plugs in the small airways.

Secretory cells in small airways express mostly mucin 5B (MUC5B), and goblet cells in large airways express mostly mucin 5AC (MUC5AC) (46). Submucosal glands in the large airways secrete mostly MUC5B. Because the mucus plugs in the small airways of humans with CF are positive for both MUC5B and MUC5AC (45), it is reasonable to believe that they may originate in part from submucosal glands in the large airways or a combination of secretory cells and goblet cells (46, 47).

CF as a chronic airway inflammatory disease is characterized by goblet cell metaplasia and increased mucus secretion (48). In healthy small airways, the mucin genes are constitutively expressed in secretory cells and mucins are continuously secreted in low amounts (46). In goblet cells, mucins are stored in granules and are released in a large amount in response to certain stimuli. Purinergic agonists are potent mucin secretagogues and result in a marked increase (>100 fold) in mucin secretion (49). Purinergic agonists exert other effects that are predicted to increase mucociliary clearance such as activation of non-CFTR chloride channels and increases in ciliary beat frequency (42). Our data indicate that excessive mucin secretion in CF small airways impairs mucociliary clearance. This finding is consistent with the ovalbumin-induced allergic goblet cell metaplasia mouse model where purinergic stimulation with adenosine triphosphate (ATP) resulted in excessive mucus occluding the airways (50).

We previously reported that a combination of acidic ASL pH and decreased liquid secretion in the lumen of submucosal glands contributes to the abnormal viscoelastic properties of mucus strands (20, 21, 47). Small airway epithelial cultures obtained from pig CF airways have decreased Cl secretion, acidic ASL pH, and increased ASL viscosity (17). In vivo measurements are not available due to difficulties accessing the small airways. However, in flattened excised small airways, we found that CF airways cleared fluorescent microspheres slower than non-CF. Moreover, after purinergic stimulation, most of the microspheres in CF pigs got stuck (51).

Inhalation deposition patterns of particles in the airways depend, in addition to the physical and chemical characteristics of the particles, on the anatomy and physiology of the respiratory tract (52). The small airways of large mammals, including humans and pigs, are similar in size to the large airways of small animals such as mice. This distinction raises the question of whether, in small animals such as mice, the deposition of small particles is like that in large mammals such as pigs and humans and whether they are equipped with the same mucus clearance mechanisms. Interestingly, mice airways lack submucosal glands (53). In the large airways of humans and pigs with CF, CFTR-mediated ${\text{HCO}}_{3}^{-}$ secretion is lacking (54). As a result, H^+^ secretion by ATPase H^+^/K^+^ transporting non-gastric alpha2 subunit (ATP12A) is unopposed and the ASL is abnormally acidic (19). In mice with CF, the absence of CFTR has little effect on ASL pH because ATP12A is not expressed in mouse airways (55). This may be a reason why CF mice do not get CF airway disease. The small airways of humans and pigs do not express ATP12A either (55). They are more like mouse airways. Thus, the lack of CFTR-mediated ${\text{HCO}}_{3}^{-}$ secretion may have little effect on ASL pH and MCC. However, we found that pigs’ small airways express a V-type proton pump ATPase (ATP6V0D) that is also expressed in the clear cells of the vas deferens (51). The localization of this pump on the cell luminal surface is pH-dependent (51). Under alkaline pH, it is translocated to the apical membrane where it results in net H^+^ secretion (51). The ASL pH in cultures of small airways from CF and non-CF pigs is alkaline enough (>7) to allow translocation of the V-ATPase to the apical membrane and, as a result, the CF small airway epithelial cultures were more acidic than their non-CF counterpart (19). Our data are consistent with an abnormal small airway MCT that may be related to abnormally acidic small airway ASL pH (51). However, these findings need to be confirmed with direct measurements in vivo and with interventions predicted to alkalinize the ASL.

Purinergic stimulation is predicted to induce Cl^−^ secretion from non-CFTR channels such as TMEM16A (56). Because of this, a P2Y receptor agonist was tested in people with CF. A phase III trial on 466 people with CF showed no clinical benefit of denufosol compared with placebo (57). Even though we have not measured ASL height in CF small airways, our data suggest that increased mucus secretion and the abnormal viscoelastic property of CF mucus outweigh the effects of increased ASL height on small airway mucociliary clearance (58).

This study has many advantages. First, this novel technique measures mucociliary clearance in vivo with high temporal and spatial resolution (59, 60). This technique allows for three-dimensional data analysis in contrast to planar data acquisition with single-photon techniques (61). In addition, the accompanying high-resolution CT scan allows for regional data analysis. Because each event is recorded and timestamped, reconstruction of the data into images with a range of time frames on the order of seconds is possible and allows capturing of fast clearance (59, 62). Second, we study newborn CF pigs with pristine lungs that lack infection, inflammation, or airway remodeling. Studying these animals in the newborn age allows us to solely evaluate the innate host defense defects at an early timepoint (19, 23). The animals are also spontaneously breathing during the procedure and are only instrumented for delivery, which allow them to maintain their own airway humidification. Studying pigs is advantageous because their respiratory physiology resembles humans. They have gland-containing proximal large airways, much like humans, and the CF porcine model reproduces all the hallmarks of CF airway disease, as seen in people with CF (2). Third, we delivered radiotracer particles of relevant size directly into the distal airways. Single-photon assays aerosolize radiotracers via a nebulizer that results in a large amount of the tracer depositing in the central large airways even when the target is the peripheral small airways. A distal delivery via a bronchoscope ensures deposition into the most distal airways.

This work also has limitations. First, this study is done on a human scanner, designed for imaging lungs in adult humans, with transfer function of the camera that is much larger than the size of newborn piglets. Because of this size discrepancy, the high-resolution CT scan also could not resolve the small airways (<200 µm airways) in the small-size newborn pigs (17). In addition, the particles were instilled into the distal airways and not aerosolized. This becomes less important as we transition this technique for use in humans. To circumvent these limitations, we used two complementary analysis schemes. A traditional concentric 3-D shell scheme with a set depth to encompass most of the small airways and a region of interest scheme extending from the main airway to the pleura. Second, these schemes are still limited. The concentric 3-D shell will also contain alveoli. The tracer trapped and retained in the alveolar space will underestimate the mucociliary clearance. The smallest airway diameter the CT scan can resolve is 2 mm. Therefore, the extended region of interest to the pleura starts from an airway with a diameter of 2 mm and will contain some airways that are considered large and are rich in submucosal glands. This will overestimate mucociliary clearance. Third, lung disease is known to be heterogeneous, and here, only a subset of the small airways is analyzed.

This work, along with previous studies, further characterizes mucus transport in small and large airways in healthy and CF pigs (20, 21, 47, 63, 64). The fast temporal aspect of PET allowed us to assess mucociliary clearance in small airways at relevant time points. Therapeutic interventions aimed at treating small airway disease in CF will benefit from this technique. Moreover, since small airways have been implicated in the pathogenesis of diseases that lack large animal models such as asthma, chronic obstructive pulmonary disease (COPD), and MUC5B promoter variant rs35705950-related idiopathic pulmonary fibrosis (IPF), it would be important to translate this methodology to study humans with these diseases (65, 66).

## DATA AVAILABILITY

All study data are included in the article and/or supporting information. Code for PET analysis is available from the corresponding author upon request.

## GRANTS

This work was funded in part by NIH, K08: HL135433 (to M.H.A.A.); NIH, R01: HL167025 (to M.H.A.A.), HL136813 (to D.A.S. and J.Z.); NIH, PPG: HL091842, HL051670 (to D.A.S. and J.Z.); and Cystic Fibrosis Foundation: ABOU20A0-KB (to M.H.A.A.), STOLTZ16XX0 (to D.A.S. and M.H.A.A.), and STOLTZ19R0 (to D.A.S. and M.H.A.A.).

## DISCLOSURES

The authors declare the following potential conflicts of interest with respect to the research, authorship, and/or publication of this article. The University of Iowa Research Foundation has licensed intellectual property related to gene-modified pigs to Exemplar Genetics. Royalties from that license are shared with D.A.S. D.A.S. has no other financial ties to Exemplar Genetics. None of the other authors has any conflicts of interest, financial or otherwise, to disclose.

## AUTHOR CONTRIBUTIONS

C.G.S., D.W.D., D.A.S., J.Z., and M.H.A.A. conceived and designed research; C.G.S., B.M.H., N.D.G., R.J.A., D.W.D., and M.H.A.A. performed experiments; C.G.S., D.W.D., J.J.S., D.A.S., J.Z., and M.H.A.A. analyzed data; C.G.S., D.W.D., J.J.S., D.A.S., J.Z., and M.H.A.A. interpreted results of experiments; C.G.S., D.A.S., J.Z., and M.H.A.A. prepared figures; C.G.S., D.A.S., J.Z., and M.H.A.A. drafted manuscript; C.G.S., B.M.H., N.D.G., R.J.A., D.W.D., J.J.S., D.A.S., J.Z., and M.H.A.A. edited and revised manuscript; C.G.S., B.M.H., N.D.G., R.J.A., D.W.D., J.J.S., D.A.S., J.Z., and M.H.A.A. approved final version of manuscript.
